# Navigating sarcoidosis: Recognizing, managing, and supporting patients in primary care

**DOI:** 10.1080/13814788.2024.2418307

**Published:** 2024-10-24

**Authors:** Marjolein Drent, Nellie Jans

**Affiliations:** a Faculty of Health, Medicine and Life Sciences, Maastricht University, Maastricht, The Netherlands; b Interstitial Lung Disease (ILD) Center of Excellence, Department of Respiratory Medicine, St. Antonius Hospital, Nieuwegein, The Netherlands; c ild care foundation research team, Ede, The Netherlands; d non-practising general practitioner, Arnhem, The Netherlands

**Keywords:** Primary care, fatigue, general practice, multisystemic disease, sarcoidosis

## Abstract

**Background:**

Sarcoidosis is a chronic multisystem inflammatory disease of unknown aetiology, characterised by noncaseating granulomas and a variable clinical presentation. Despite its global distribution, sarcoidosis is relatively rare, with the highest prevalence in northern Europe. This poses challenges for primary care physicians due to its broad spectrum of symptoms, from organ-specific manifestations to general complaints like fatigue and concentration difficulties.

**Objectives:**

This article aims to provide primary care physicians with practical tools for the early recognition and management of sarcoidosis, emphasising their role in monitoring disease progression and providing supportive care.

**Methods:**

Key strategies for diagnosis and management are reviewed, focusing on holistic patient care addressing both somatic and psychosocial aspects of the disease.

**Results:**

Early recognition, careful monitoring of disease progression, and individualised treatment plans are crucial. Pharmacotherapy is not always required and should be carefully balanced. The role of supportive, patient-centered counseling is illustrated with two cases.

**Conclusion:**

Primary care physicians play a critical role in managing sarcoidosis, particularly in early recognition and monitoring. Given the absence of standardised treatment protocols, a flexible, holistic approach that includes psychosocial support is essential. This article provides a practical framework for general practitioners to address the challenges of sarcoidosis management and improve patient outcomes.

## KEY MESSAGES

Sarcoidosis an inflammatory disorder that can affect any organ, is rarely seen in primary care practice.

The clinical presentation and course are divers and unpredictable, with fatigue being the most pronounced symptom.

Treatment in primary and secondary care focuses on both somatic and psychosocial aspects, including encouraging a healthy lifestyle.

## Introduction

Sarcoidosis is a rather rare inflammatory disorder, in which cells of the immune system accumulate in granulomas [1–3]. The clinical expression, natural history, and prognosis of sarcoidosis are highly variable and its course is often unpredictable. Clinical manifestations vary with the organs involved. Approximately 90% of patients with sarcoidosis experience involvement of the lungs and/or mediastinal lymph nodes. The disease can also affect other organs and tissues such as the skin, eyes, and heart. Remission occurs in more than one-half of patients within 3 years of diagnosis, and within 10 years in two-thirds, with few or no remaining consequences [2]. Interpretation of the severity of the sarcoidosis can be complicated by its heterogeneity. Genetic predisposition, certain antigens like silica (a crystalline mineral found in sand, quarts and cat litter) and pesticides, but also stressful events, play a part in its development [1,2,4].

Sarcoidosis is a multisystem disorder, and the involvement of various organs varies with patients’ ethnic background. The incidence and prevalence of sarcoidosis and its clinical presentation vary greatly across geographical regions and between the sexes and different ethnicities and age groups [5]. Sarcoidosis can develop in people of all ages. The average age of onset is 40–55 years of age, with a younger peak age at diagnosis in men (30–50 years of age) than in women (50–60 years of age), a pattern that is confirmed by several reports in different regions with a second peak among women after menopause [1,2,6]. Its course is often more severe among people with a dark skin [1,2]. The global prevalence and incidence is difficult to calculate because of different diagnostic criteria and clinical heterogenicity. The annual incidence of sarcoidosis varies worldwide between regions. The rates are lowest in Asian countries, including China, Japan and South Korea (1-5 case per 100,000), higher in North America and Australia (5–10 cases per 100,000) and highest in Northern European (Scandinavian) countries (up to 40 cases per 100,000) [1,6]. As sarcoidosis is a uncommon disorder most primary care physicians have limited experience in the management of sarcoidosis [3].

## Clinical presentation

**Figure 1.**
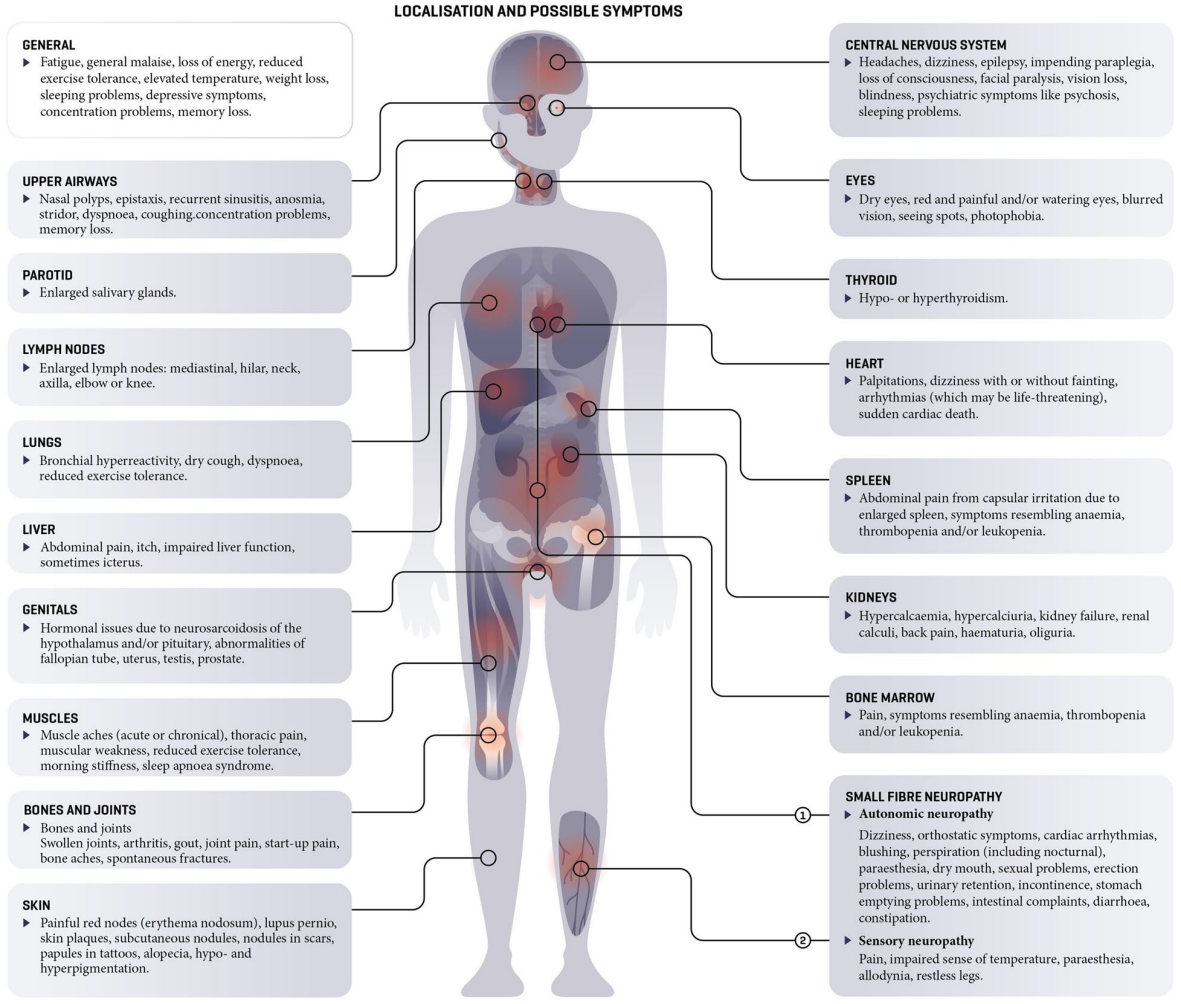
Organ-specific and general symptoms of sarcoidosis.

The clinical presentation of sarcoidosis is highly variable, usually depending on the location of the granulomatous inflammation as shown in Figure 1 [2,7–9]. In the majority of patients, the disease involves the lungs or lymph nodes, which may not necessarily lead to symptoms. Involvement of the heart, liver or kidneys can cause arrhythmias, hepatitis or kidney failure. Granulomatous inflammation in the central nervous system can result in a facial nerve palsy, epilepsy, and even paraplegia [9]. Muscle involvement, rather common but often not recognised, can cause loss of power in the extremities, as well as sleeping problems [9,10]. Sarcoidosis of the skin can be manifest in many forms, like erythema nodosum and, nodules in scars and tattoos [11]. Other possible consequences are hypercalcaemia, hypercalciuria and kidney stones. The eyes may also be involved, which can lead to uveitis, cataract, dry eyes, glaucoma or even blindness. There is also an elevated risk of osteoporosis, whether caused by the disorder itself or by its treatment (with steroids).

Sarcoidosis frequently has a non-specific clinical presentation, featuring symptoms like cognitive impairments, memory loss, weight loss or pain. The most commonly reported non-specific symptom is fatigue [2,8,12,13]. In part of the cases, the pain symptoms and autonomic nervous system dysfunctions can be explained by small fibre neuropathy (see also Figure 1) [7,8,14]. The impact of sarcoidosis on the quality of life of both patients and their partners is large [15].

## Diagnostic work-up

Sarcoidosis is a diagnosis by exclusion [16]. Patients usually present with non-specific complaints, including fatigue, and thorough history-taking is required to recognise the coherent pattern of symptoms [7]. It is important to take an underlying systemic disorder into consideration, especially if the patient has had multiple, long-lasting symptoms. For instance, the pattern of symptoms in a post-COVID-19 syndrome can closely resemble that of sarcoidosis [17]. If sarcoidosis is suspected, the physical examination focuses mainly on the skin, while fatigue can be assessed using a short online questionnaire, the Fatigue Assessment Scale [12,13] presented in Table 1. This questionnaire can be used to assess fatigue in any situation the presence of fatigue is assumed, it measures both physical and mental symptoms, and is available in more than 25 languages [12]. This scale can also be useful in tracking fatigue over time.

**Table 1.** Fatigue Assessment Scale (FAS) [12,13]. The following ten statements refer to how you usually feel. Per statement you can choose one out of five answer categories, varying from Never to Always. Please circle the answer to each question that is applicable to you. Please give an answer to each question, even if you do not have any complaints at the moment. 1 = Never, 2 = Sometimes (about monthly or less); 3 = Regularly (about a few times a month); 4 = Often (about weekly) and 5 = Always (about every day).

	Never	Sometimes	Regularly	Often	Always
1. I am bothered by fatigue.	1	2	3	4	5
2. I get tired very quickly.	1	2	3	4	5
3. I don’t do much during the day.	1	2	3	4	5
4. I have enough energy for everyday life.	1	2	3	4	5
5. Physically, I feel exhausted.	1	2	3	4	5
6. I have problems to start things.	1	2	3	4	5
7. I have problems to think clearly.	1	2	3	4	5
8. I feel no desire to do anything.	1	2	3	4	5
9. Mentally, I feel exhausted.	1	2	3	4	5
10. When I am doing something, I can concentrate quite well.	1	2	3	4	5

Scores on questions 4 and 10 should be recoded (1 = 5, 2 = 4, 3 = 3, 4 = 2, 5 = 1). Subsequently, the total FAS score can be calculated by summing the scores on all questions (the recoded scores for question 4 and 10). The sum of questions 3 and 6–9 indicates mental fatigue, and the sum of the questions 1, 2, 4, 5 and 10 indicates physical fatigue. The minimal score is 10 the maximal score is 50. Based on large representative samples of the Dutch population, the cut-off score of the FAS is 21, i.e. scores of ≥22 are considered to represent substantial fatigue. A change in the FAS score of four points is considered to be clinically relevant (minimal clinically important different) [13].

Reproduced with the permission of the ild care foundation (www.ildcare.nl).

Depending on the findings of history-taking and the clinical presentation, laboratory tests can be ordered to exclude other causes of fatigue, like thyroid problems and anaemia. Normal blood values do not, however, exclude sarcoidosis, and conversely, there is no single laboratory test that can definitively confirm the diagnosis of sarcoidosis. Lab tests used in the diagnostic work-up and follow-up of patients with sarcoidosis are angiotensin converting enzyme (ACE) and serum-soluble interleukin-2 receptor (sIL2R) [16].

Although chest X-ray examinations will not always provide certainty in cases of suspected sarcoidosis either, as a chest X-ray showing no abnormalities does not exclude sarcoidosis, the primary care physician is encouraged to obtain a chest radiograph in patients where sarcoidosis should be considered. Chest X-rays may show enlarged mediastinal lymph nodes, which in combination with arthritis, erythema nodosum and fever are classical manifestations of Löfgren’s syndrome (see also Figures 2a and 2b). In that case, no further histological or cytological examinations are necessary. In other cases, histological confirmation is necessary [2,16]. If the patient has cardiac symptoms, like arrhythmias, an ECG is recommended. In a secondary care setting, a PET-scan can be used to establish the inflammatory activity and the extent of organ involvement, including cardiac involvement, while an MRI can be used to assess any cardiac or neurological manifestations [2,16].

**Figure 2a.**
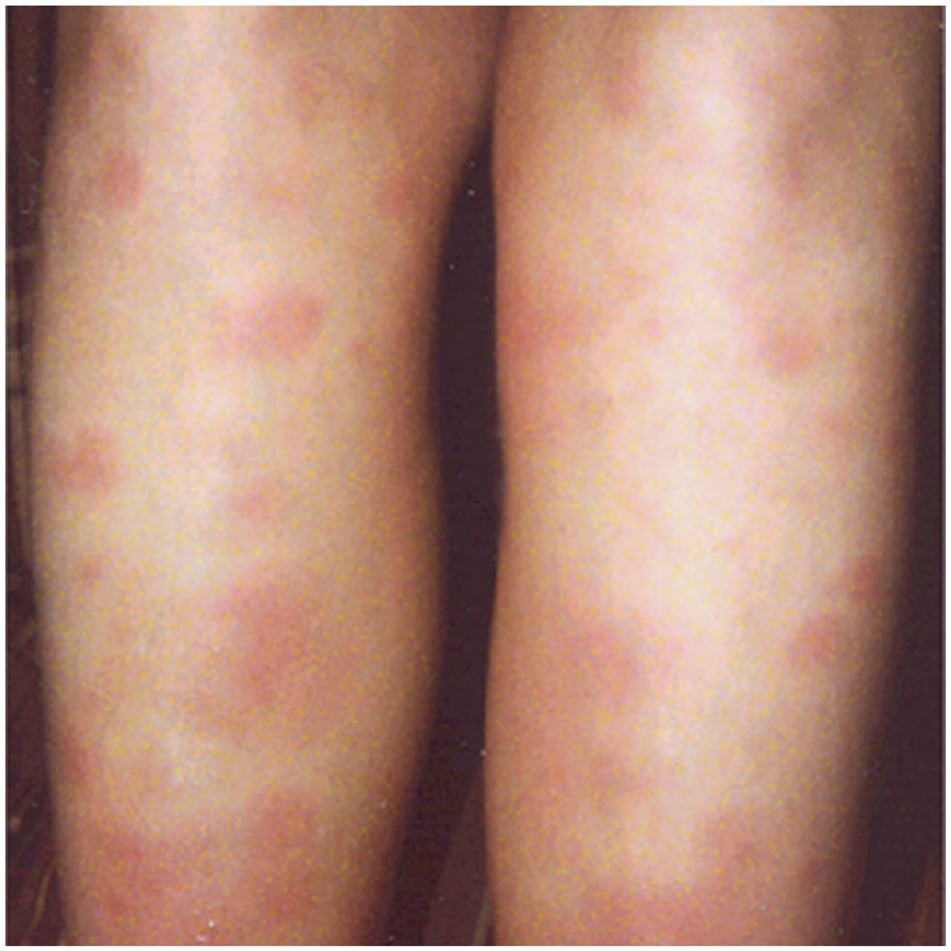
Image of the skin of both lower legs of a 36-year-old woman (patient A) showing red raised patches (erythema nodosum).

**Figure 2b.**
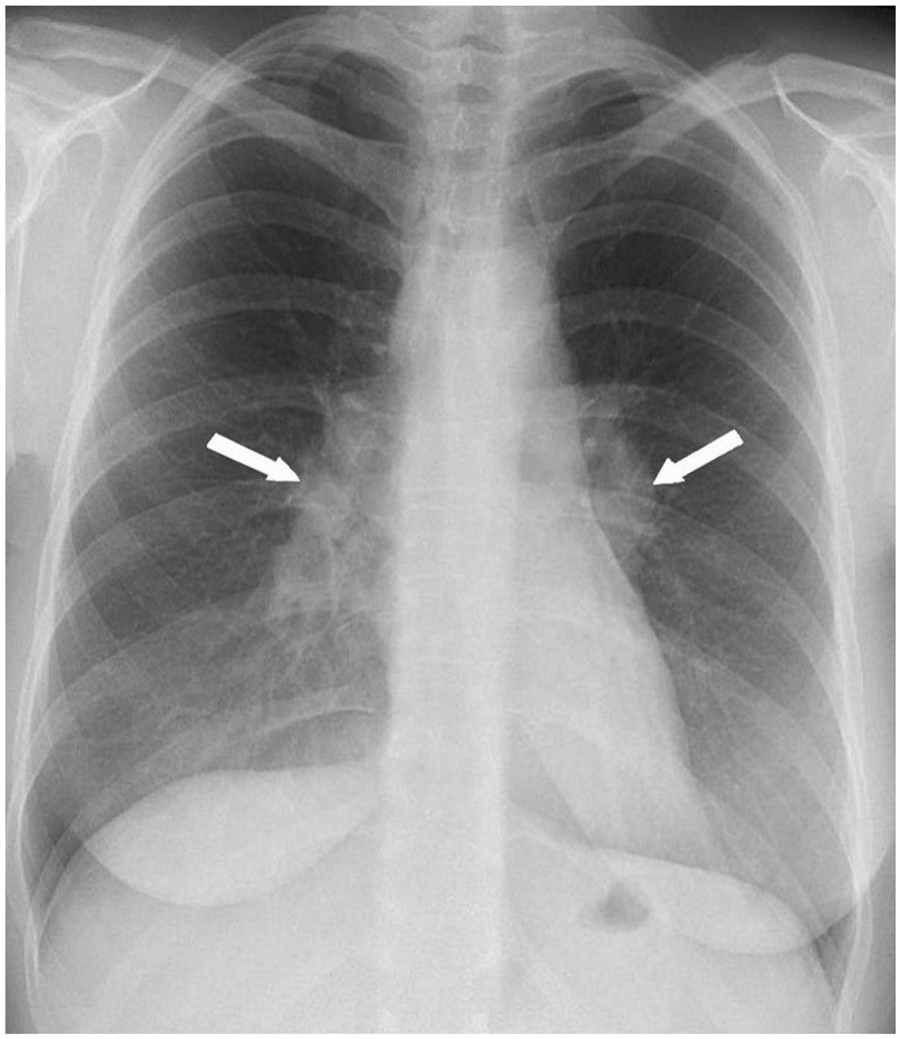
Chest X-ray of patient A showing bilateral lymphadenopathy (arrows).

## Treatment

The major goals in treating sarcoidosis are lowering the morbidity and mortality risk and improving quality of life [2,7,18]. Sarcoidosis patients do not always need to be treated with drugs, even during active disease episodes [3,18,19]. Lifestyle interventions, like exercise programmes, a healthy diet and mindfulness may be sufficient [7,19,20]. For patients with Löfgren’s syndrome, it is often enough to treat the symptoms, such as non-steroidal anti-inflammatory drugs (NSAIDs) in case of painful joints, as long as there are no functional impairments [2,18]. Drug treatment in a secondary care setting is indicated when organs are damaged, to prevent serious or even life-threatening complications. Absolute indications for systemic therapy include substantially reduced lung function, and manifestations of the disease in the eyes, central nervous system or heart. Relative indications include exercise-related dyspnoea, extreme fatigue or pain, if these symptoms substantially reduce the patient’s quality of life [2,3,15,18].

In general, there are three options of drug treatment. The drug of first choice is the immunomodulatory drug prednisone [2,18]. If this provides insufficient relief, or there is a contraindication to corticosteroids, the next option is a combination of methotrexate and folic acid or azathioprine [2,18]. A Dutch study is currently examining whether prednisone could be replaced by methotrexate as the drug of first choice, in view of the latter drug’s more favourable adverse effects profile [21]. A third option, if methotrexate has insufficient effect, are is to start are TNF-alpha inhibitors like infliximab and adalimumab or biosimilars [2,18]. The drugs currently used to treat various manifestations of sarcoidosis are liable to cause adverse events and sometimes substantial co-morbidities. It is important to consider the possibility of drug-induced damage in sarcoidosis, especially if the clinical situation deteriorates after the introduction of a particular drug [22]. These comorbidities further increase the burden of this disease, and also affect quality of life [15]. A major challenge for clinicians in primary and secondary care is to rethink and reconfigure therapeutic approaches to the disease, including shared decision making with patients to improve adherence to treatment, optimise its benefits, and reduce the risk of adverse effects [8,23].

## Course and prognosis

The course of the disease is difficult to predict. Periods in which the patient feels relatively well are alternated with periods of worsening symptoms [2]. Acute-onset sarcoidosis usually has a favourable course: 70% of patients had a full recovery after 2–3 years, with or without medication, while 25% develop chronic disease, complicated by fibrosis potentially leading to irreversible organ failure and even death (see also the Box with two examples of various clinical presentations). The mortality rate is 5%. Non-specific symptoms, especially fatigue and cognitive failure, can persist for a long time, even after objective abnormalities have disappeared [2,7,8,24]. This has a huge impact on a patient’s quality of life.

Since sarcoidosis occurs particularly among young people, the disease can lead to problems with their job or employer and in turn, socio-economic problems [25–28]. About 30% of the patients inevitably have to reduce their working hours because of fatigue and other symptoms associated with sarcoidosis [27,29].

Box. Two examples of different clinical presentation:

**Patient A** is a 36-year-old woman with worsening symptoms of extreme fatigue, painful ankle joints and fever over the last two weeks. She has painful, hard, red raised patches on the skin of both lower legs and painful, red, swollen ankle joints (Figure 2a). Blood tests show elevated CRP (30 mg/L), and a chest X-ray shows bilateral lymphadenopathy (Figure 2b). The acute nature and characteristic clinical presentation indicated Löfgren’s syndrome. According to international guidelines, this characteristic clinical presentation does not require histological confirmation [16]. The diagnosis was confirmed by a pulmonologist. Since there were no functional impairments, non-steroidal anti-inflammatory drugs (NSAIDs) were prescribed to relieve the symptoms. Moreover, the patient was referred back to the primary care physician. After 6 months, she was free of symptoms.

**Patient B** is a 40-year-old man with increasing fatigue and loss of concentration and memory. He has a young family and a busy, responsible job. Exploratory examination by the patient’s family doctor reveals nothing specific, and she considers a burn-out. The patient then breaks his collarbone in a bicycle accident. An X-ray shows the fracture, but also diffuse abnormalities in both lungs. The man is referred to a pulmonologist who did a lung function test revealing a low diffusing capacity for carbon monoxide (60%), as well as a low forced vital capacity (70%). In view of his severely reduced lung function, he was prescribed oral prednisone, 20 mg a day. After 6 months his clinical situation has been improved, as well as his lung function tests (just over 10%). He continued to be followed up by the pulmonologist.

## Supportive primary care

Sarcoidosis can affect any organ in the body, leading to highly variable disease presentations, symptoms, and limitations. It is a relatively uncommon condition that clinicians, particularly in primary care, seldom encounter. The low specificity of its symptoms and the general unfamiliarity with sarcoidosis among primary care physicians and other clinicians can lead to delays in diagnosis [3]. The initial exploratory examination often fails to reveal anything specific, and further diagnostics easily stray into alternative explanations like ‘stress-related problems’ or ‘menopause’. To promote early recognition and avoid such delays, it is essential for clinicians, including primary care physicians, to listen carefully, reflect, seek advice, and acknowledge the crucial role patients themselves play in the diagnostic process [30].

Worldwide Sarcoidosis Centres of Excellence, which consist of multidisciplinary teams of specialised medical and paramedical professionals, provide leadership, best practices, research, support and training for sarcoidosis patients and professionals including primary care physicians (see also www.wasog.org). In general, they are advised to refer patients to a medical specialist to establish the diagnosis and the extend of the disease and to determine whether patients need to be referred for treatment in a sarcoidosis centre.

Some disease consequences and complications may be difficult to verify [7,30]. Multidisciplinary management involving sarcoidosis experts and allied health professionals can help in understanding and managing symptoms as a consequence of sarcoidosis [15,23,30]. Supportive care, such as physical training and mindfulness for patients with fatigue or deconditioning, is often beneficial, in terms of improving quality of life [19,20,28]. The primary care physician plays a key role in recognising deterioration and adverse drug reactions [3].

Providing suitable information to patients, appropriate holistic guidance, including understanding their limitations and receiving social support, can significantly improve the rehabilitation [15].

## Conclusion

Sarcoidosis is a multisystem disorder. For a primary care physician, establishing the diagnosis starts with considering sarcoidosis by listening to the patient, extensive history taking, and referring to a pulmonologist or other organ specialist in case of a sense of alarm. If the disease takes a chronic course, the primary care physician should be aware of the highly variable nature of its clinical presentation, deterioration of the disease, and drug side effects. The pulmonologist usually coordinates the multidisciplinary management and tailored care of sarcoidosis patients.
